# Gender-Specific Associations Between Tobacco and Tobacco-Free Nicotine Use and Symptoms of Anxiety and Depression in Swedish Adolescents

**DOI:** 10.1177/1179173X261455075

**Published:** 2026-05-22

**Authors:** Johanna Andersson, Malin Hansson, Mia Ericson, Louise Adermark

**Affiliations:** 1 Department of Pharmacology, Sahlgrenska Academy, 195564 University of Gothenburg Institute of Neuroscience and Physiology, Gothenburg, Sweden; 2 Institute of Health and Care Sciences, 70712 University of Gothenburg Sahlgrenska Academy, Gothenburg, Sweden; 3 Research and Development Primary Healthcare, Region Västra Götaland, Gothenburg, Sweden; 4 Department of Psychiatry and Neurochemistry, Sahlgrenska Academy, 195564 University of Gothenburg Institute of Neuroscience and Physiology, Gothenburg, Sweden

**Keywords:** e-cigarettes, HADS, nicotine, nicotine pouches, psychiatric morbidity, teenagers

## Abstract

**Background:**

The use of non-combustible tobacco-free nicotine (TFN) products has increased markedly among adolescents. Although often perceived as less harmful than traditional tobacco products, nicotine use has been associated with psychological distress.

**Aim:**

This study examined whether TFN use is associated with symptoms of anxiety and depression in adolescents, whether these associations differ from those of traditional tobacco products, and whether gender identity affects the outcome.

**Methods:**

Adolescents aged 17 to 19 years were invited to complete an online questionnaire assessing nicotine use and psychological distress, measured using the Hospital Anxiety and Depression Scale (HADS). Data were analyzed using chi-square tests and logistic regression models.

**Results:**

A total of 3730 adolescents participated, of whom 30% reported nicotine use. TFN use was more common than tobacco use, while dual use was more prevalent among boys. Both TFN and tobacco use were associated with increased odds of anxiety or depression symptoms, with similar effect sizes across product types. However, association were partly gender-dependent. Among girls using nicotine, 80% reported anxiety symptoms, with higher frequency of use associated with greater risk. Among boys, nicotine use was associated with depressive symptoms, and dual product use was associated with an increased risk of psychological distress.

**Conclusion:**

Nicotine use is associated with psychological distress regardless of product type. Girls may be particularly vulnerable to anxiety-related symptoms, while dual use appears especially risky among boys. These findings highlight the need for gender-sensitive prevention strategies targeting dual use and the progression to regular nicotine use.

## Introduction

Tobacco smoking is a major risk factor for morbidity and mortality, accounting for millions of deaths each year.^1^ Beyond its well-established role in somatic disease, smoking is also associated with psychiatric morbidity and is overrepresented among individuals with anxiety and affective disorders.^2^ However, the relationship between nicotine exposure and psychological distress is complex, and both the underlying mechanisms and direction of this association remain incompletely understood. Nicotine use may be initiated as a form of self-medication to alleviate negative affect,^3^ yet repeated exposure has also been shown to induce persistent neurophysiological and molecular alterations that may affect brain function and emotional processing.^4-6^ Importantly, the association between nicotine exposure and psychiatric morbidity appears to be moderated by sex and gender.^2,6,7^ Evidence suggests that nicotine influences reward-related neural circuits in a sex-specific manner, and women often report greater relief from negative affect following nicotine use compared with men.^8,9^ These findings indicate that nicotine may exert differential effects on the brain, potentially contributing to sex-specific mental health outcomes. Further investigating the role of sex- and gender is therefore warranted.

While the global prevalence of cigarette smoking has declined over recent decades,^10^ the use of tobacco-free nicotine (TFN) products, such as electronic cigarettes (e-cigarettes) and nicotine pouches, has increased, particularly among adolescents. In Sweden, smoking has traditionally been more common among girls, whereas boys more frequently have used Swedish snus, a moist oral tobacco product similar to nicotine pouches.^11^ However, following the introduction of TFN products, patterns of nicotine use among Swedish youth have shifted, with nicotine pouches gaining substantial popularity among young girls.^12^ Recent Swedish school surveys indicate that more than 50 % of both boys and girls in lower secondary school have tried snus products (including Swedish snus and nicotine pouches), and an even higher proportion have experimented with e-cigarettes.^12^ However, it remains unclear whether adolescents use traditional tobacco products and TFN products exclusively or in combination. Further research aimed to improve the understanding of nicotine use patterns among Swedish adolescents is thus needed.

While TFN products often is marketed as an harm reduction product compared to tobacco cigarettes, their high nicotine content may still pose risks, not only for cardiovascular disorders but also for the developing brain.^11,13^ Similar to cigarette smoking, the use of TFN products has been associated with increased risk for psychiatric morbidity.^2,14,15^ However, beyond the effects of nicotine itself, cigarette smoke contains additional compounds that may influence neurotransmitter systems involved in mood regulation,^16^ potentially further increasing the risk of psychiatric symptoms. At the same time, TFN generally contains flavors and additives, which may have neuroactive effects themselves.^17^ Therefore, research comparing traditional tobacco products and TFN in relation to psychological distress is important to further outline putative risks associated with nicotine use.

In addition to characterizing patterns of nicotine use among Swedish adolescents, this study aimed to examine whether the use of TFN products is associated with psychological distress and increased risk of anxiety or depression, whether these associations depend on gender identity, and whether relative risks differed from traditional tobacco products. Considering both the similarities and differences between traditional tobacco and TFN products, as well as previously reported sex- and gender-specific effects, we hypothesized that nicotine use would be associated with higher risk of both anxiety and depressive symptoms regardless of product type, but more strongly among girls. To test this hypothesis, we conducted an online survey in which adolescents reported their use of nicotine products and completed the Hospital Anxiety and Depression Scale (HADS) to assess symptoms of psychological distress with special emphasis on anxiety and depression.

## Methods

### Study Design

A cross-sectional study was conducted to investigate the association between tobacco and TFN use and symptoms of psychiatric morbidity among Swedish adolescents. The study was approved by the Swedish Ethical Review Authority (202305459-01).

### Setting

Data were collected at upper secondary schools (17-19 years old) in central and southern Sweden during late fall 2023. School health care personnel posted an advertisement for the study containing a QR code connected to an on-line questionnaire. Questions addressed the use of nicotine containing products, alcohol consumption and substance use, as well as psychiatric health.

### Participants

Participants that decided to scan the QR code and provided consent to participate in the study (n=3730) were further investigated for eligibility. Specifically, the inclusion criteria were i) born between 2004-2006 (i.e., expected to study at the Swedish upper secondary school during the spring of 2023, age ∼17-19), ii) gender identity being either male or female, and ii) provide valid responses on variables addressing use of nicotine containing products, risk consumption of alcohol, as well as items addressing symptoms of psychiatric morbidity. Individuals reporting gender identity “other” was excluded due to the low number of individuals, which would not only reduce the power in statistical testing, but potentially also infringe the anonymity of study participants. Questions are further outlined in supplemental material.

### Need for Consent Regarding Individuals Under 18 Years of Age

In Sweden, research subjects who have reached the age of 15 but not 18 must be informed about and consent to the research themselves. Under the Swedish Ethical Review Act (SFS 2003:460), consent requirements for minors are not based solely on chronological age, but on the individual’s capacity and maturity to understand what participation in the research entails. A dual consent from both minors and guardians for individuals under 18 years of age is thus not a requirement. This is a matter of a legally established right of self-determination for the young person themselves. It is not uncommon for research concerning the relevant age group to focus on issues that are specific to that particular group. It may concern intimate and privacy-sensitive information within the framework of the sphere that, even in a confidentiality context, is protected even from the view of guardians. In order to ensure the young person’s right to make their own independent decision about their participation, guardians should not be informed about the planned research in these cases.

### Variables

#### Use of Nicotine Containing Products

Tobacco use was addressed using the question “*Do you use tobacco?”* (Yes/No). Participants who reported tobacco use were further asked, “*How often do you use tobacco*? with response options: once a month or less often/several times a month/several times a week/daily, consistent with previous studies.^11^ Use of tobacco-free nicotine (TFN) products (e.g., nicotine pouches, e-cigarettes) was assessed using parallel questions: “Do you use tobacco-free nicotine products?” (Yes/No) and “How often do you use tobacco-free nicotine products?” with the same response categories. No specific product examples were provided in the survey. Questions and response alternatives are further presented in Supplemental material.

Participants reporting exclusive use of either tobacco of TFN products were categorized as Exclusive use. Those reporting use of both tobacco and TFN products were classified as Dual use. Participants reporting no nicotine use was set as the reference category.

##### Frequency of Nicotine Use

Nicotine use was separated into two groups, either using more sporadic (*Sporadic use)*, which included participants using less than weekly (once a month or less often/several times a month), or using regularly (*Regular use*), which entailed individuals using any form of nicotine most days of the month (every week/daily). For dual use, the highest frequency of use reported for either product was used in the analysis.

#### Symptoms of Mental Health Problems

To examine the association between nicotine use and symptoms of anxiety or depression, study participants completed the Hospital Anxiety and Depression Scale (HADS), a 14-item questionnaire addressing symptoms of anxiety and depression.^18^ The HADS comprises two subscales, anxiety (HADS-A), and depression (HADS-D), each consisting of seven items with total scores ranging from 0 to 21, where higher scores indicate greater symptom severity. Clinically relevant symptoms of anxiety or depression were defined as a score of ≥8 points on the respective subscales.^18^ The total score (HADS-T) was used as a measure of overall psychological distress. Previous research suggests that a cutoff of ≥13 provides an optimal balance between sensitivity and specificity for general clinical use.^19^ The HADS has been validated in Swedish populations^20^ as well as among adolescents.^21^ In the present study, the total scale and both subscales demonstrated acceptable internal consistency (Cronbach’s alpha: HADS-T, α= 0.85; HADS-A, α= 0.83; HADS-D, α= 0.72).

#### Covariates

##### Age

Participants were asked in which year they were born. An approximate age was calculated by subtracting the year of birth from the year of data collection (2023).

##### Gender

Participants were asked: Which is your gender identity?, with response alternatives: Female, Male, Other. The gender identity was examined rather than biological sex. However, due to few responses in the category “other” (32 individuals in total), only males and females were included in the analysis.

##### Risk-Consumption of Alcohol

Risk-consumption of alcohol was measured using AUDIT-C, the first three items of the Alcohol Use Disorders Identification Test (AUDIT).^22,23^ Each item is responded to on a 5-grade scale, ranging from 0-4 points. The responses on each item are summarized into a total AUDIT-C score ranging from 0-12 points (α=0.67). Individuals that did not report any alcohol consumption for the past 12 months were assigned a score of 0 points. An AUDIT-C score of 3≥points, which has been demonstrated to have a high sensitivity and specificity for identifying at-risk consumption of alcohol among adolescents,^23^ and the dichotomous outcome (risk consumption: yes/no) was used when adjusting for hazardous consumption. Both AUDIT and AUDIT-C have been validated in a Swedish setting.^24^

##### Substance Use

Participants were asked if they had ever engaged in unauthorized use of prescription medicine (benzodiazepines, pain medications containing opioids, and central stimulants). They were also asked if they had ever used cannabinoids, amphetamine, opiates, hallucinogens, ecstasy or cocaine. Responses on all items were summarized into a dichotomous variable (yes or no).

### Statistical Analysis

Data analyses were conducted using IBM SPSS Statistics (version 29). Group differences in categorical variables were assessed using chi-square tests. Associations between different forms of nicotine exposure and symptoms of psychological distress, including either anxiety or depression, were examined using separate logistic regression models. In addition to univariate analyses, all models were adjusted for gender identity, risk alcohol use, and substance use. To assess potential effect modification by gender, fully adjusted models including an interaction term between gender identity and type of nicotine product used were also fitted.

A one-way ANOVA was conducted to examine differences in HADS-T scores (dependent variable) across nicotine use groups (independent variable: no use, sporadic use, regular use). Post hoc pairwise comparisons were conducted using Bonferroni correction. In all analyses, the significance level was set to <0.05. Results are presented as Mean ± SD, or Odd Ratio (OR) with corresponding 95% confidence intervals (CI), unless anything else is clearly stated. Full models for the data are presented in supplemental information.

## Results

### Study Population

The analytical sample consisted of 2978 respondents, with significantly higher proportion of girls than boys (χ^2^(1, n=2978) = 9.25, p=0.002) (Table 1). There were no gender differences in mean age or risk consumption of alcohol, but a greater proportion of girls reported having engaged in substance use compared to boys (Table 1). Mental health problems were more prevalent among girls, with a higher proportion of girls scoring ≥8 points on both the HADS-A and HADS-D (χ^2^(1, n=2978) = 397.56, p<0.001; χ^2^(1, n=2978) = 24.44, p<0.001) (Table 1).

**Table 1.** Study Population

​	Total	Girls	Boys	p
Sample size (n)	2978	1572	1406	​
%	​	**52.8**	**47.2**	**0.002**
AUDIT-C≥3p				
%	46.8	47.5	46.1	0.435
Substance use				
%	17.5	**18.8**	**16.0**	**0.047**
Age				
Mean (SD)	17.9 (0.8)	17.9 (0.8)	17.9 (0.8)	0.721
HADS-A ≥8				
%	51.2	**68.4**	**31.9**	**<0.001**
HADS-D ≥8				
%	25.6	**29.3**	**21.4**	**<0.001**
HADS-T ≥13				
%	50.7	**63.9**	**36.1**	**<0.001**

Bold values indicate statistically significant results.

### Use of Nicotine Containing Products – Product Type and Frequency of Use

Nicotine use was common among study participants, with nearly one third reporting use of any nicotine-containing product and no overall gender difference (Table 2). Tobacco use was more prevalent among boys than girls (17.1% vs. 11.7%, χ^2^(1, n = 2978) = 17.93, p < 0.001), but the use of TFN products did not differ significantly (girls: 26.5% vs. boys: 23.5%, p = 0.060) (Table 2).

**Table 2.** Prevalence of Nicotine Product Use

​	Total % (n)	Girls % (n)	Boys % (n)	p
Nicotine use*	29.9 (891)	30.5 (480)	29.2 (411)	0.438
Regular nicotine use#	69.4 (618)	**62.1 (298)**	**77.9 (320)**	**<0.001**
Tobacco use*	14.3 (425)	**11.7 (184)**	**17.1 (241)**	**<0.001**
TFN use*	25.1 (746)	26.5 (416)	23.5 (330)	0.060
Exclusive tobacco use#	16.3 (145)	13.3 (64)	19.7 (81)	0.056
Exclusive TFN use#	52.3 (466)	**61.7 (296)**	**41.4 (170)**	**<0.001**
Dual use#	31.4 (280)	**25.0 (120)**	**38.9 (160)**	**0.003**

*In relation to total number of study participants.

#: In relation to participants reporting any use of nicotine.

Bold values indicate statistically significant results.

To further examine patterns of use, participants reporting any nicotine use were categorized into exclusive or dual use of tobacco and TFN products. Within these subgroups, exclusive TFN use was more prevalent among girls (χ^2^(1, n = 2553) = 19.25, p < 0.001), whereas boys were more likely to report dual use (χ^2^(1, n = 2367) = 8.86, p = 0.003) (Table 2). Among participants reporting tobacco use, 34.1% reported exclusive use, while 62.5% of individuals reporting TFN use exclusively used TFN. No significant gender differences were observed in exclusive tobacco use.

Among individuals reporting use of any nicotine-containing product, regular use was more common than sporadic use (69.4% vs. 30.6%; χ^2^(1, n = 891) = 133.59, p < 0.001). Furthermore, boys reported regular nicotine use more frequently than girls (Table 2). The distribution of use by product type (tobacco and/or TFN) and frequency is presented in Table 3. Regular use of TFN products was the most prevalent pattern, whereas the combination of regular tobacco use and sporadic TFN use was the least common.

**Table 3.** Frequency of Use Among Individuals Reporting Nicotine Use

Frequency of tobacco use	Frequency of TFN use		
	No use	Sporadic	Regular
No use	-	19.6 % (n=175)	32.7 % (n=291)
Sporadic (less than weekly)	4.6 % (n=41)	6.4 % (n=57)	7.2 % (n=64)
Regular (several times a week)	11.7 % (n=104)	2.6 % (n=23)	15.3 % (n=136)

### Association Between Nicotine Use and Symptoms of Anxiety and Depression

The associations between nicotine use and symptoms of anxiety and depression (HADS-T ≥13, HADS-A ≥8 and HADS-D ≥8, respectively) were assessed using logistic regression. In univariate models, all forms of nicotine use (Exclusive tobacco use, Exclusive TFN use and Dual use) were associated with increased odds for psychological distress, as well as symptoms of anxiety and depression, with odds ratios ranging between 1.40 and 2.47 (Table 4). After adjusting for gender identity, risk consumption of alcohol, and substance use, all forms of nicotine use were associated with increased odds for psychological distress and depressive symptoms, whereas exclusive tobacco use no longer remained a significant predictor for symptoms of anxiety (Table 4). Full models are presented in supplemental material.

**Table 4.** Associations Between Nicotine Use and Symptoms of Psychological Distress

​	HADS-T (≥13) Odds ratio (95% CI)		HADS-A (≥8) Odds ratio (95% CI)		HADS-D (≥8) Odds ratio (95% CI)	
	Univariate	Adjusted^a^	Univariate	Adjusted^a^	Univariate	Adjusted^a^
Exclusive tobacco use (n=2232) (n=145)^b^	**1.85 (1.31-2.62) p<0.001**	**1.76 (1.21-2.56) p=0.003**	**1.46 (1.04-2.05) p=0.029**	1.38 (0.94-2.02) p=0.099	**2.47 (1.75-3.49) p<0.001**	**2.44 (1.68-3.53) p<0.001**
Exclusive TFN use (n=2553) (n=466)^b^	**1.90 (1.55-2.34) p<0.001**	**1.66 (1.32-2.08) p<0.001**	**1.92 (1.56-2.36) p<0.001**	**1.54 (1.22-1.95) p<0.001**	**1.40 (1.12-1.76) p=0.003**	**1.43 (1.12-1.82) p=0.005**
Dual use (n=2367) (n=280)^b^	**1.77 (1.38-2.29)** **<0.001**	**1.62 (1.21-2.16) p=0.001**	**1.59 (1.23-2.04) p<0.001**	**1.45 (1.08-1.95) p=0.014**	**1.64 (1.25-2.15) p<0.001**	**1.52 (1.11-2.06) p=0.008**

Reference category was set to no nicotine use in all models.

^a^Adjusted for, gender, risk consumption of alcohol and substance use.

^b^Absolute number of participants reporting use of relevant nicotine product.

HADS-T (≥13): Symptoms of overall psychological distress.

HADS-A (≥8): Symptoms of anxiety.

HADS-D (≥8): Symptoms of depression.

Bold: Statistically significant

### Associations Between Nicotine Use and Symptoms of Anxiety and Depression by Gender

To assess whether the association between nicotine use and psychological distress was modified by gender, a full model including an interaction term between “nicotine use” and gender identity was fitted for HADS-T ≥13. The analysis showed that gender identity modified the association (p=0.011), and hence full models for HADS-A and HADS-D including interaction terms between gender identity and the different forms of nicotine product use were fitted. Results showed a significant interaction only between “Exclusive TFN” use and gender, with respect to symptoms of anxiety (p=0.007) (data not shown).

**Figure 1.**
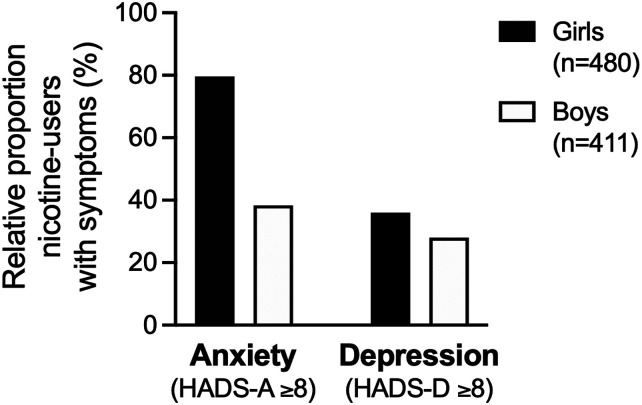
Symptoms of anxiety and depression in relation to nicotine use Bar graph illustrating the proportion of participants meeting criteria for clinically relevant symptoms of anxiety or depression among individuals reporting nicotine use. Notably, a very high proportion of girls using nicotine reported symptoms of anxiety. n=number of responses

To further examine the role of gender identity for the association between nicotine use and symptoms of anxiety and depression, models were fitted separately for individuals identifying themselves as girls and boys. After adjusting for covariates, dual use was associated with HADS-T ≥13 in boys, whereas any form of nicotine use predicted elevated scores in girls. Exclusive tobacco or TFN use was a significant predictor of depressive symptoms regardless of gender, even after controlling for risky alcohol and substance use (Table 5). However, after adjustment, dual use remained a significant predictor of depressive symptoms only in boys (Table 5). Regarding anxiety, dual use predicted symptoms selectively in boys, whereas exclusive use of tobacco or TFN predicted symptoms only in girls (Table 5). Full models are presented in Supplemental material. Notably, among girls reporting any nicotine use, 80.0% had HADS-A scores ≥8 and 36.0% had depressive symptoms (HADS-D ≥8; Figure 1). Among boys using nicotine, 38.4% had HADS-A scores ≥8, and 28.0% had HADS-D scores ≥8 (Figure 1).

**Table 5.** Associations Between Nicotine Use and Symptoms of Psychological Distress Stratified by Gender

​	HADS-T (≥13) Odds ratio (95% CI)		HADS-A (≥8) Odds ratio (95% CI)		HADS-D (≥8) Odds ratio (95% CI)	
	Univariate	Adjusted^a^	Univariate	Adjusted^a^	Univariate	Adjusted^a^
	Exclusive tobacco						
Girls n=1156 (n=64)^b^	**3.46 (1.79-6.69) p<0.001**	**2.38 (1.20-4.72) p=0.013**	**3.10 (1.56-6.15) p=0.001**	**2.16 (1.06-4.39) p=0.033**	**2.97 (1.79-4.94) p<0.001**	**2.34 (1.36-4.02) p=0.002**
Boys n=1076 (n=81)^b^	**1.63 (1.03-2,57) p=0.037**	1.57 (0.97-2.53) p=0.065	1.28 (0.80-2.07) p=0.305	1.11 (0.67-1.83) p=0.685	**2.30 (1.42-3.73) p<0.001**	**2.55 (1.52-4.26) p<0.001**
Exclusive TFN						
Girls n=1388 (n=296)^b^	**2.19 (1.64-2.93) p<0.001**	**2.0 (1.47-2.62) p<0.001**	**2.21 (1.63-3.01) p<0.001**	**1.95 (1.41-2.69) p<0.001**	1.32 (0.99-1.74) p=0.051	**1.36 (1.01-1.83) p=0.046**
Boys n=1165 (n=170)^b^	1.29 (0.92-1.81) p=0.136	1.27 (0.88-1.83) p=0.20	1.29 (0.91-1.82) p=0.144	1.13 (0.78-1.65) p=0.512	1.38 (0.94-2.03) p=0.100	**1.55 (1.02-2.37) p=0.041**
Dual use						
Girls n=1212 (n=120)^b^	**2.36 (1.52-3.66) p<0.001**	**1.69 (1.06-2.69) p=0.029**	**1.98 (1.27-3.09) p=0.003**	1.40 (0.87-2.25) p=0.165	**1.68 (1.12-2.48) p=0.010**	1.35 (0.87-2.08) p=0.181
Boys n=1155 (n=160)^b^	**1.89 (1.35-2.64) <0.001**	**1.68 (1.15-2.46) p=0.008**	**1.94 (1.38-2.73) p<0.001**	**1.53 (1.04-2.24) p=0.031**	**1.76 (1.20-2.56) p=0.004**	**1.75 (1.12-2.71) p=0.012**

Reference category was set to no nicotine use in all models.

^a^Adjusted for risk consumption of alcohol, and substance use.

^b^Absolute number of participants reporting use of nicotine.

HADS-T (≥13): Symptoms of overall psychological distress.

HADS-A (≥8): Symptoms of anxiety.

HADS-D (≥8): Symptoms of depression.

Bold values indicate statistically significant results.

### Frequency of Nicotine Use in Relation to HADS-T

**Figure 2.**
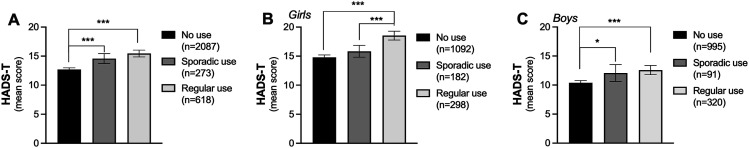
Frequency of nicotine use in relation to HADS-T score A) Overall psychological distress (HADS-T score) was significantly higher among participants reporting nicotine use. B) Among girls, regular nicotine use (several times a week) was associated with higher HADS-T scores compared to sporadic use (less than weekly). C) Among boys, both sporadic and regular nicotine use were associated with higher HADS-T scores, with no difference observed between levels of use frequency. Data are mean values with 95% confidence intervals. n=number of responses. * p<0.05, *** p<0.001

To assess if the frequency of nicotine use could influence the association with mental health problems, participants reporting nicotine use were categorized into sporadic (monthly or less often) and regular (daily or several times a week). Comparison of mean HADS-T scores across individuals reporting no use, sporadic nicotine use and regular nicotine use showed that mean HADS-T score differed significantly between groups (F (2) =40.99, η^2^_p_=0.027). Post hoc analyses indicated that both sporadic and regular nicotine use were associated with higher HADS-T scores compared to no use (no use vs. sporadic use, p<0.001; no use vs. regular use, p<0.001), while no significant difference was observed between sporadic and regular use (p=0.252) (Figure 2A).

In gender-stratified analyses, significant differences between exposure groups were observed for both girls (F (2) =35.40, p<0.001) and boys (F (2) =15.88, p<0.001). Among girls, regular nicotine use was associated with higher HADS-T scores compared to both no use and sporadic use (no use vs. sporadic use: p = 0.179; no use vs. regular use: p < 0.001; sporadic use vs. regular use: p < 0.001) (Figure 2B). In contrast, among boys, higher HADS-T scores were observed for both sporadic and regular use compared to no use (no use vs. sporadic use: p = 0.046; no use vs. regular use: p < 0.001), with no difference between sporadic and regular use (p = 1.00) (Figure 2C).

## Discussion

The aim of this study was to examine the association between nicotine use and symptoms of psychological distress, with a particular focus on anxiety and depression among Swedish adolescents. Specifically, we sought to determine whether these associations differed by type of nicotine product (tobacco or TFN) and by gender identity. Our findings indicate that nicotine use, regardless of whether the product contains tobacco, is associated with symptoms of anxiety and depression, although the patterns of association is partially dependent on gender identity. Overall, the associations were more consistent among girls than boys, with a substantial proportion of girls using nicotine reporting clinically relevant symptoms of anxiety. Among girls, more frequent nicotine use was associated with a higher risk of psychological distress, suggesting a dose–response–like pattern. In contrast, among boys, the frequency of use did not affect risk score, but dual use of nicotine products was associated with increased risk of psychological distress. Taken together, these findings suggest that the association between nicotine use and psychiatric morbidity is largely independent of tobacco content but moderated by gender. The high prevalence of psychological distress among girls who use nicotine is particularly concerning and highlights the need for increased awareness among school health services.

Nicotine use was common among study participants, consistent with findings from other school-based surveys of Swedish adolescents.^12^ Use was also frequent, with the majority of individuals reporting consumption several times per week. Approximately one-third of participants who reported nicotine use indicated concurrent use of both tobacco and TFN products. Although the overall prevalence of nicotine use did not differ by gender, exclusive use of TFN was more prominent among girls, while dual use was more common among boys. In addition, boys were more likely to report regular use, in line with findings from other Nordic countries.^25^ These gender-specific patterns highlight the need for tailored prevention and intervention strategies. Among boys, efforts may benefit from focusing on reducing multiproduct use, limiting the appeal of products such as Swedish snus, and increasing awareness of the risks associated with frequent and daily consumption. For girls, prevention strategies may be more effective if they target the transition from occasional to regular use, while also emphasizing the addictive potential of nicotine, potential gender-specific vulnerabilities, and the risks associated with nicotine exposure during pregnancy.^26^

A high percentage of participants reported symptoms of psychological distress, with a notably higher prevalence among girls. These findings are consistent with the increasing rates of psychological and psychosomatic symptoms reported among Swedish adolescents and underscore the need for early detection and intervention.^27,28^ It is important to note that, although HADS is a useful screening tool with reasonable sensitivity and specificity,^19^ it is not a diagnostic instrument. The total score may not always precisely classify patients into distinct diagnostic categories of anxiety or depression.^29^ Consequently, further clinical assessment is required to validate the high prevalence of psychiatric morbidity observed in this sample.

The data presented here indicate that nicotine use is associated with significantly increased odds of reporting symptoms of psychological distress, including anxiety and depression. This association was observed across product types (tobacco and TFN), with similar effect sizes across all nicotine-using groups. These findings suggest that TFN products should not be considered to confer harm-reduction benefits with respect to the risk of psychological distress. Notably, dual use of both tobacco and TFN further increased the risk among boys, highlighting a cumulative effect that may exacerbate vulnerability to mental health problems. Taken together, these results underscore that any form of nicotine use, and particularly dual use, is associated with adverse psychological outcomes, emphasizing the need for targeted prevention and intervention strategies that address all types of nicotine products rather than assuming harm reduction from TFN alternatives.

Although causal inferences cannot be drawn from the cross-sectional design of this study, a substantial body of evidence has consistently shown an association between nicotine dependence to an increased incidence of psychiatric disorders. Nicotine use may therefore contribute to the onset or exacerbation of such conditions.^4,30-35^ Longitudinal studies further support this relationship, demonstrating that nicotine dependence is associated with the persistence of psychiatric and substance use disorders among individuals with pre-existing conditions.^36^ While nicotine may provide short-term symptom relief in some individuals with psychiatric disorders,^37^ chronic use does not appear to improve the likelihood of remission.^36^ Mechanistic evidence also supports an association between nicotine exposure and psychiatric morbidity. Neuroimaging studies implicate nicotinic acetylcholine receptors (nAChRs) in the pathophysiology of disorders such as post-traumatic stress disorder (PTSD),^38^ and preclinical studies indicate that these receptors play a role in modulating depression-like behavior.^39-41^ Beyond receptor-level effects, repeated nicotine exposure may induce neurophysiological and biochemical changes in the brain, potentially increasing vulnerability to the development of psychiatric illness.^6,42-44^

The patterns of association between nicotine use and symptoms of psychological distress differed by gender identity. Symptoms of anxiety were overrepresented among girls using nicotine, an association not observed among boys reporting exclusive use of either tobacco or TFN products. However, dual use was associated with increased odds also among boys. Gender-specific associations between nicotine use and psychiatric morbidity are supported by previous studies, which suggest that women who use nicotine have a higher risk of reporting mental health problems compared with men.^7,45-47^ These differences may partly reflect variation in motives of use. Adolescent girls are more likely to use nicotine for emotional regulation, while boys are more likely to cite reasons connected to reward and reinforcing effects.^48^ Experimental and clinical studies further indicates that women may experience stronger stress-relieving and mood-regulating effects from nicotine than men, potentially increasing the likelihood of use in response to anxiety and negative mood.^9^ However, evidence from both human and animal research suggest that nicotine exerts partially gender-specific effects on the brain, including alterations in neural circuits involved in emotional regulation.^8,49-53^ Prospective studies in adolescents and young adults have also shown that occasional smoking predicts later panic attacks in women but not men, further indicating a heightened vulnerability to anxiety-related symptoms among women who use nicotine.^54^ Taken together, these findings underscore the importance of considering sex- and gender-specific mechanisms in understanding the relationship between nicotine use and psychological distress. Further research is needed to clarify these associations and to better characterize the underlying neurobiological pathways.

This study has several limitations. First, the types of tobacco or TFN product used were not addressed in the survey. It is thus not possible to outline if the outcome depended on the route of administration. In addition, the online survey did not provide examples of products within each category. Although TFN products is a well-established concept in Sweden, there is a risk of misclassification and inaccurate reporting. For instance, nicotine-free e-cigarettes may be classified as nicotine containing, and heated tobacco products may be misconceived as tobacco free. Secondly, although socioeconomic status is known to be associated with both nicotine use and psychiatric morbidity, analyses were not adjusted for this factor. However, in the Swedish context, a high proportion of adolescents report having a similar economic situation to their peers, and previous research suggests that socioeconomic status does not substantially influence the association between tobacco use and psychiatric morbidity.^55^ Another limitation is the absence of a priori power calculations, which may affect the interpretability of the findings, particularly in subgroup analyses. It should further be acknowledged that the present study assessed gender identity but did not examine biological sex. As such, the identified gender differences should not be interpreted as purely biological, since they may also capture the influence of socially constructed gender roles, identities, and normative expectations. Finally, the cross-sectional design limits the ability to determine the directionality or causality of the observed associations.

## Conclusion

The findings of this study indicate that patterns of nicotine use are partly gender-specific, with boys reporting more frequent use and a higher prevalence of dual use of tobacco and TFN products. Nicotine use was associated with increased odds of psychological distress, independent of product type, although the pattern differed by gender identity. A high proportion of girls using any nicotine-containing product reported symptoms of anxiety, whereas this association was observed only among boys reporting dual use. Taken together, these findings suggest that TFN products confer risks comparable to those of tobacco with respect to psychological distress and should not be assumed to represent a safer alternative in this context. They further underscore the importance of gender-sensitive prevention and intervention strategies targeting all forms of nicotine use. Finally, the high prevalence of psychological distress among Swedish adolescents is concerning and highlights the need for continued research to better understand the underlying drivers of this significant public health challenge.

## Supplemental Material

Supplemental material - Gender-Specific Associations Between Tobacco and Tobacco-Free Nicotine Use and Symptoms of Anxiety and Depression in Swedish Adolescents Supplemental material for Gender-Specific Associations Between Tobacco and Tobacco-Free Nicotine Use and Symptoms of Anxiety and Depression in Swedish Adolescents by Johanna Andersson, Malin Hansson, Mia Ericson, and Louise Adermark in Tobacco Use Insights.

## Data Availability

The data that support the findings of this study are available from Professor Mia Ericson but restrictions apply to the availability of these data, which were used under license for the current study, and so are not publicly available. Data are however available from the authors upon reasonable request and with permission of Professor Mia Ericson.*
