# Supplementary material for: Gender-Specific Associations Between Tobacco and Tobacco-Free Nicotine Use and Symptoms of Anxiety and Depression in Swedish Adolescents
Source: Tob Use Insights. 2026 May 22;19:1179173X261455075. doi: 10.1177/1179173X261455075 (PMC13197629; doi:10.1177/1179173X261455075)
Supplement: Supplemental material - Gender-Specific Associations Between Tobacco and Tobacco-Free Nicotine Use and Symptoms of Anxiety and Depression in Swedish Adolescents [file sj-pdf-1-tui-10.1177_1179173X261455075.pdf]

Supplemental information

Gender-specific associations between tobacco and tobacco-free nicotine use and symptoms of anxiety and depression in Swedish adolescents

Johanna Andersson[1], Malin Hansson[2,3], Mia Ericson*[4], Louise Adermark*[1]

[1]Institute of Neuroscience and Physiology, Department of Pharmacology, Sahlgrenska Academy, University of Gothenburg, Gothenburg, Sweden

[2]Institute of Health and Care Sciences, Sahlgrenska Academy, University of Gothenburg, Gothenburg, Sweden

[3]Region Västra Götaland, Research and Development Primary Healthcare, Sweden.

[4]Institute of Neuroscience and Physiology, Department of Psychiatry and Neurochemistry, Sahlgrenska Academy, University of Gothenburg, Gothenburg, Sweden

Questions included in the survey

Gender

Which is your gender identity?

- Female
- Male
- Other

Nicotine and Tobacco use

Do you use tobacco?

- Yes
- No

How often do you use tobacco?

- Once a month or less
- Several times a month
- Several times a week
- Daily

Do you use tobacco-free nicotine products?

- Yes
- No

How often do you use tobacco-free nicotine products?

- Once a month or less
- Several times a month
- Several times a week
- Daily

Hospital Anxiety and Depression Scale (HADS)

Answer options to each question:

- Mostly
- Often
- Sometimes
- Not at all

Questions:

- I feel tense and nervous:
- I still appreciate things I used to appreciate:
- I have a feeling that something terrible is going to happen:
- I can laugh and see the fun in things:
- I worry about things:
- I feel in a good mood:
- I can sit still and feel relaxed:
- Everything feels sluggish:
- I feel anxious, like I have "butterflies in my stomach":
- I have lost interest in how I look:
- I feel restless:
- I look forward to things with joy:
- I get sudden panic attacks:

I can appreciate a good book, a TV or radio program:

Alcohol consumption (AUDIT-C)

How often do you have a drink containing alcohol?

Never (0)
Monthly or less (1)
2-4 times a month (2)
2-3 times a week (3)
4 or more times a week (4)

How many drinks containing alcohol do you have on a typical day when you are drinking?

1 or 2 (0)
3 or 4 (1)
5 or 6 (2)
7 to 9 (3)
10 or more (4)

How often do you have six or more drinks on one occasion?

Never (0)
Less than monthly (1)
Monthly (2)
Weekly (3)
Daily or almost daily (4)

Substance use

Have you ever used prescription medicines (such as benzodiazepines, pain medications containing opioids, or central stimulants) in an unauthorized manner.

Yes
No

Have you ever used cannabinoids, amphetamine, opiates, hallucinogens, ecstasy or cocaine?

Yes
No

Full models for data presented in Table 4:
HADS-A≥8p
Exclusive tobacco

Variables in the Equation

		B	S.E.	Wald	df	Sig.	Exp(B)	95% C.I.for EXP(B) Lower	Upper
Step 1[a]	Exclusive_tobacco(1)	.322	.195	2.718	1	.099	1.380	.941	2.024
	Gender_dikotom(1)	-1.476	.092	257.063	1	<.001	.228	.191	.274
	AUDIT_C_risk(1)	.124	.096	1.659	1	.198	1.132	.937	1.366
	Substance_use(1)	.772	.145	28.398	1	<.001	2.163	1.629	2.873
	Constant	.445	.072	38.188	1	<.001	1.560		

a. Variable(s) entered on step 1: Exclusive_tobacco, Gender_dikotom, AUDIT_C_risk, Substance_use.

Exclusive TFN

Variables in the Equation

		B	S.E.	Wald	df	Sig.	Exp(B)	95% C.I.for EXP(B) Lower	Upper
Step 1[a]	Exclusive_tobaccofree(1)	.433	.120	13.050	1	<.001	1.542	1.219	1.950
	Gender_dikotom(1)	-1.543	.087	314.867	1	<.001	.214	.180	.253
	AUDIT_C_risk(1)	.062	.092	.456	1	.500	1.064	.889	1.274
	Substance_use(1)	.772	.133	33.531	1	<.001	2.165	1.667	2.812
	Constant	.496	.070	50.511	1	<.001	1.642		

a. Variable(s) entered on step 1: Exclusive_tobaccofree, Gender_dikotom, AUDIT_C_risk, Substance_use.

Dual use

Variables in the Equation

		B	S.E.	Wald	df	Sig.	Exp(B)	95% C.I.for EXP(B) Lower	Upper
Step 1[a]	Dual_users(1)	.371	.151	6.062	1	.014	1.450	1.079	1.948
	Gender_dikotom(1)	-1.439	.090	258.538	1	<.001	.237	.199	.283
	AUDIT_C_risk(1)	.111	.095	1.385	1	.239	1.118	.929	1.346
	Substance_use(1)	.817	.135	36.477	1	<.001	2.264	1.737	2.952
	Constant	.428	.071	36.491	1	<.001	1.534		

a. Variable(s) entered on step 1: Dual_users, Gender_dikotom, AUDIT_C_risk, Substance_use.

HADS-D≥8p
Exclusive tobacco

Variables in the Equation

		B	S.E.	Wald	df	Sig.	Exp(B)	95% C.I.for EXP(B)	
								Lower	Upper
Step 1[a]	Exclusive_tobacco(1)	.890	.189	22.153	1	<.001	2.436	1.681	3.530
	Gender_dikotom(1)	-.438	.103	18.169	1	<.001	.645	.528	.789
	AUDIT_C_risk(1)	-.417	.110	14.333	1	<.001	.659	.531	.818
	Substance_use(1)	.680	.141	23.341	1	<.001	1.973	1.498	2.599
	Constant	-.979	.079	154.807	1	<.001	.376		

a. Variable(s) entered on step 1: Exclusive_tobacco, Gender_dikotom, AUDIT_C_risk, Substance_use.

Exclusive TFN

Variables in the Equation

		B	S.E.	Wald	df	Sig.	Exp(B)	95% C.I.for EXP(B)	
								Lower	Upper
Step 1[a]	Exclusive_tobaccofree	.354	.125	8.059	1	.005	1.425	1.116	1.819
	Gender_dikotom(1)	-.436	.096	20.533	1	<.001	.646	.535	.781
	AUDIT_C_risk(1)	-.359	.103	12.196	1	<.001	.698	.571	.854
	Substance_use(1)	.608	.128	22.640	1	<.001	1.837	1.430	2.361
	Constant	-.987	.076	170.601	1	<.001	.373		

a. Variable(s) entered on step 1: Exclusive_tobaccofree, Gender_dikotom, AUDIT_C_risk, Substance_use.

Dual use

Variables in the Equation

		B	S.E.	Wald	df	Sig.	Exp(B)	95% C.I.for EXP(B)	
								Lower	Upper
Step 1[a]	Dual_users(1)	.416	.157	7.028	1	.008	1.515	1.114	2.060
	Gender_dikotom(1)	-.413	.100	17.157	1	<.001	.662	.545	.805
	AUDIT_C_risk(1)	-.388	.109	12.727	1	<.001	.679	.548	.840
	Substance_use(1)	.894	.131	46.922	1	<.001	2.446	1.894	3.159
	Constant	-1.029	.078	174.973	1	<.001	.357		

a. Variable(s) entered on step 1: Dual_users, Gender_dikotom, AUDIT_C_risk, Substance_use.

HADS-T≥13p

Exclusive tobacco

Variables in the Equation

		B	S.E.	Wald	df	Sig.	Exp(B)	95% C.I.for EXP(B)	
								Lower	Upper
Step 1[a]	Exclusive_tobacco(1)	.564	.192	8.648	1	.003	1.757	1.207	2.559
	Gender_dikotom(1)	1.063	.089	141.505	1	<.001	2.894	2.429	3.448
	AUDIT_C_risk(1)	-.012	.093	.017	1	.897	.988	.823	1.186
	Substance_use(1)	.759	.140	29.226	1	<.001	2.136	1.622	2.813
	Constant	-.798	.075	113.417	1	<.001	.450		

a. Variable(s) entered on step 1: Exclusive_tobacco, Gender_dikotom, AUDIT_C_risk, Substance_use.

Exclusive TFN

Variables in the Equation

		B	S.E.	Wald	df	Sig.	Exp(B)	95% C.I.for EXP(B)	
								Lower	Upper
Step 1[a]	Exclusive_tobaccofree(1)	.505	.116	18.923	1	<.001	1.657	1.320	2.080
	Gender_dikotom(1)	1.137	.084	182.480	1	<.001	3.117	2.643	3.676
	AUDIT_C_risk(1)	-.092	.089	1.050	1	.305	.913	.766	1.087
	Substance_use(1)	.726	.128	31.989	1	<.001	2.067	1.607	2.658
	Constant	-.808	.073	123.667	1	<.001	.446		

a. Variable(s) entered on step 1: Exclusive_tobaccofree, Gender_dikotom, AUDIT_C_risk, Substance_use.

Dual use

Variables in the Equation

		B	S.E.	Wald	df	Sig.	Exp(B)	95% C.I.for EXP(B)	
								Lower	Upper
Step 1[a]	Dual_users(1)	.481	.148	10.580	1	.001	1.618	1.211	2.162
	Gender_dikotom(1)	1.055	.087	146.718	1	<.001	2.873	2.422	3.408
	AUDIT_C_risk(1)	-.061	.093	.438	1	.508	.941	.785	1.128
	Substance_use(1)	.894	.133	45.325	1	<.001	2.444	1.884	3.170
	Constant	-.791	.074	114.406	1	<.001	.453		

a. Variable(s) entered on step 1: Dual_users, Gender_dikotom, AUDIT_C_risk, Substance_use.

Full models for data presented in Table 5.
HADS-A≥8p

Exclusive tobacco
Variables in the Equation

Gender_dikotom			B	S.E.	Wald	df	Sig.	Exp(B)	95% C.I.for EXP(B)	
									Lower	Upper
0 Female	Step 1[a]	Exclusive_tobacco(1)	.771	.362	4.544	1	.033	2.162	1.064	4.392
		AUDIT_C_risk(1)	.210	.131	2.580	1	.108	1.234	.955	1.595
		Substance_use(1)	.823	.210	15.404	1	<.001	2.278	1.510	3.436
		Constant	.392	.080	24.004	1	<.001	1.479		
1 Male	Step 1[a]	Exclusive_tobacco(1)	.103	.255	.165	1	.685	1.109	.673	1.827
		AUDIT_C_risk(1)	.040	.143	.076	1	.782	1.040	.785	1.378
		Substance_use(1)	.700	.207	11.476	1	<.001	2.013	1.343	3.019
		Constant	-.973	.087	125.161	1	<.001	.378		

a. Variable(s) entered on step 1: Exclusive_tobacco, AUDIT_C_risk, Substance_use.

Exclusive TFN
Variables in the Equation

Gender_dikotom			B	S.E.	Wald	df	Sig.	Exp(B)	95% C.I.for EXP(B)	
									Lower	Upper
0 Female	Step 1[a]	Exclusive_tobaccofree(1)	.668	.165	16.361	1	<.001	1.950	1.411	2.694
		AUDIT_C_risk(1)	.167	.123	1.836	1	.175	1.182	.928	1.504
		Substance_use(1)	.856	.195	19.196	1	<.001	2.355	1.605	3.454
		Constant	.404	.078	26.548	1	<.001	1.497		
1 Male	Step 1[a]	Exclusive_tobaccofree(1)	.125	.190	.429	1	.512	1.133	.780	1.645
		AUDIT_C_risk(1)	-.065	.140	.217	1	.641	.937	.712	1.233
		Substance_use(1)	.771	.190	16.487	1	<.001	2.161	1.490	3.135
		Constant	-.946	.085	123.148	1	<.001	.388		

a. Variable(s) entered on step 1: Exclusive_tobaccofree, AUDIT_C_risk, Substance_use.

Dual use
Variables in the Equation

Gender_dikotom			B	S.E.	Wald	df	Sig.	Exp(B)	95% C.I.for EXP(B)	
									Lower	Upper
0 Female	Step 1[a]	Dual_users(1)	.336	.242	1.925	1	.165	1.400	.870	2.250
		AUDIT_C_risk(1)	.202	.129	2.459	1	.117	1.224	.951	1.576
		Substance_use(1)	.854	.200	18.283	1	<.001	2.348	1.588	3.473
		Constant	.392	.079	24.351	1	<.001	1.479		
1 Male	Step 1[a]	Dual_users(1)	.422	.195	4.670	1	.031	1.525	1.040	2.235
		AUDIT_C_risk(1)	.005	.141	.001	1	.971	1.005	.762	1.325
		Substance_use(1)	.796	.186	18.340	1	<.001	2.218	1.540	3.193
		Constant	-.972	.086	127.578	1	<.001	.378		

a. Variable(s) entered on step 1: Dual_users, AUDIT_C_risk, Substance_use.

HADS-D≥8p
Exclusive tobacco

Variables in the Equation

Gender_dikotom			B	S.E.	Wald	df	Sig.	Exp(B)	95% C.I.for EXP(B) Lower	Upper
0 Female	Step 1[a]	Exclusive_tobacco(1)	.849	.276	9.430	1	.002	2.337	1.359	4.017
		AUDIT_C_risk(1)	-.363	.143	6.462	1	.011	.695	.525	.920
		Substance_use(1)	.793	.179	19.524	1	<.001	2.209	1.554	3.140
		Constant	-1.015	.087	136.100	1	<.001	.363		
1 Male	Step 1[a]	Exclusive_tobacco(1)	.936	.262	12.706	1	<.001	2.549	1.524	4.264
		AUDIT_C_risk(1)	-.486	.174	7.793	1	.005	.615	.437	.865
		Substance_use(1)	.497	.233	4.544	1	.033	1.644	1.041	2.597
		Constant	-1.374	.096	203.086	1	<.001	.253		

a. Variable(s) entered on step 1: Exclusive_tobacco, AUDIT_C_risk, Substance_use.

Exclusive TFN

Variables in the Equation

Gender_dikotom			B	S.E.	Wald	df	Sig.	Exp(B)	95% C.I.for EXP(B) Lower	Upper
0 Female	Step 1[a]	Exclusive_tobaccofree(1)	.306	.153	3.999	1	.046	1.358	1.006	1.832
		AUDIT_C_risk(1)	-.238	.130	3.344	1	.067	.788	.611	1.017
		Substance_use(1)	.633	.160	15.624	1	<.001	1.884	1.376	2.579
		Constant	-1.032	.085	148.026	1	<.001	.356		
1 Male	Step 1[a]	Exclusive_tobaccofree(1)	.440	.216	4.159	1	.041	1.553	1.017	2.371
		AUDIT_C_risk(1)	-.556	.170	10.659	1	.001	.574	.411	.801
		Substance_use(1)	.588	.215	7.491	1	.006	1.801	1.182	2.744
		Constant	-1.366	.095	207.216	1	<.001	.255		

a. Variable(s) entered on step 1: Exclusive_tobaccofree, AUDIT_C_risk, Substance_use.

Dual use

Variables in the Equation

Gender_dikotom			B	S.E.	Wald	df	Sig.	Exp(B)	95% C.I.for EXP(B) Lower	Upper
0 Female	Step 1[a]	Dual_users(1)	.298	.223	1.788	1	.181	1.347	.871	2.083
		AUDIT_C_risk(1)	-.292	.141	4.300	1	.038	.746	.566	.984
		Substance_use(1)	.954	.170	31.334	1	<.001	2.596	1.859	3.626
		Constant	-1.064	.087	148.512	1	<.001	.345		
1 Male	Step 1[a]	Dual_users(1)	.561	.223	6.325	1	.012	1.753	1.132	2.714
		AUDIT_C_risk(1)	-.524	.172	9.288	1	.002	.592	.423	.829
		Substance_use(1)	.822	.205	16.130	1	<.001	2.274	1.523	3.396
		Constant	-1.403	.096	213.049	1	<.001	.246		

a. Variable(s) entered on step 1: Dual_users, AUDIT_C_risk, Substance_use.

HADS-T≥13p
Exclusive tobacco

Variables in the Equation

Gender_dikotom			B	S.E.	Wald	df	Sig.	Exp(B)	95% C.I.for EXP(B) Lower	Upper
0 Female	Step 1[a]	Exclusive_tobacco(1)	.869	.349	6.203	1	.013	2.384	1.203	4.723
		AUDIT_C_risk(1)	.140	.127	1.217	1	.270	1.151	.897	1.477
		Substance_use(1)	.905	.201	20.163	1	<.001	2.471	1.665	3.668
		Constant	.181	.079	5.309	1	.021	1.199		
1 Male	Step 1[a]	Exclusive_tobacco(1)	.449	.244	3.393	1	.065	1.566	.972	2.525
		AUDIT_C_risk(1)	-.175	.140	1.560	1	.212	.839	.638	1.105
		Substance_use(1)	.595	.205	8.407	1	.004	1.813	1.213	2.710
		Constant	-.711	.083	73.851	1	<.001	.491		

a. Variable(s) entered on step 1: Exclusive_tobacco, AUDIT_C_risk, Substance_use.

Exclusive TFN

Variables in the Equation

Gender_dikotom			B	S.E.	Wald	df	Sig.	Exp(B)	95% C.I.for EXP(B) Lower	Upper
0 Female	Step 1[a]	Exclusive_tobaccofree(1)	.693	.156	19.658	1	<.001	1.999	1.472	2.716
		AUDIT_C_risk(1)	.084	.119	.502	1	.478	1.088	.862	1.374
		Substance_use(1)	.799	.181	19.474	1	<.001	2.224	1.559	3.171
		Constant	.211	.077	7.564	1	.006	1.235		
1 Male	Step 1[a]	Exclusive_tobaccofree(1)	.240	.186	1.675	1	.196	1.272	.884	1.831
		AUDIT_C_risk(1)	-.315	.137	5.241	1	.022	.730	.558	.956
		Substance_use(1)	.735	.189	15.158	1	<.001	2.086	1.441	3.019
		Constant	-.681	.081	70.347	1	<.001	.506		

a. Variable(s) entered on step 1: Exclusive_tobaccofree, AUDIT_C_risk, Substance_use.

Dual use

Variables in the Equation

Gender_dikotom			B	S.E.	Wald	df	Sig.	Exp(B)	95% C.I.for EXP(B) Lower	Upper
0 Female	Step 1[a]	Dual_users(1)	.523	.239	4.795	1	.029	1.686	1.056	2.692
		AUDIT_C_risk(1)	.138	.126	1.206	1	.272	1.148	.897	1.469
		Substance_use(1)	.923	.192	23.025	1	<.001	2.517	1.726	3.669
		Constant	.180	.078	5.329	1	.021	1.198		
1 Male	Step 1[a]	Dual_users(1)	.519	.194	7.132	1	.008	1.681	1.148	2.461
		AUDIT_C_risk(1)	-.299	.140	4.567	1	.033	.742	.564	.976
		Substance_use(1)	.894	.186	23.055	1	<.001	2.445	1.697	3.522
		Constant	-.703	.082	73.570	1	<.001	.495		

a. Variable(s) entered on step 1: Dual_users, AUDIT_C_risk, Substance_use.
